# NF-*κ*B signaling and its relevance to the treatment of mantle cell lymphoma

**DOI:** 10.1186/s13045-018-0621-5

**Published:** 2018-06-15

**Authors:** Swathi Balaji, Makhdum Ahmed, Elizabeth Lorence, Fangfang Yan, Krystle Nomie, Michael Wang

**Affiliations:** Department of Lymphoma/Myeloma, University of Texas MD Anderson Cancer Center, 1515 Holcombe Blvd. Unit 0429, Houston, TX 77030-4009 USA

**Keywords:** NF-*κ*B, Mantle cell lymphoma, Canonical pathway, Non-canonical pathway

## Abstract

Mantle cell lymphoma is an aggressive subtype of non-Hodgkin B cell lymphoma that is characterized by a poor prognosis determined by Ki67 and Mantle Cell International Prognostic Index scores, but it is becoming increasingly treatable. The majority of patients, especially if young, achieve a progression-free survival of at least 5 years. Mantle cell lymphoma can initially be treated with an anti-CD20 antibody in combination with a chemotherapy backbone, such as VR-CAP (the anti-CD20 monoclonal antibody rituximab administered with cyclophosphamide, doxorubicin, and prednisone) or R-CHOP (the anti-CD20 monoclonal antibody rituximab administered with cyclophosphamide, doxorubicin, vincristine, and prednisone). While initial treatment can facilitate recovery and complete remission in a few patients, many patients experience relapsed or refractory mantle cell lymphoma within 2 to 3 years after initial treatment. Targeted agents such as ibrutinib, an inhibitor of Bruton’s tyrosine kinase, which has been approved only in the relapsed setting, can be used to treat patients with relapsed or refractory mantle cell lymphoma. However, mantle cell lymphoma cells often acquire resistance to such targeted agents and continue to survive by activating alternate signaling pathways such as the PI3K-Akt pathway or the NF-*κ*B pathways.

NF-*κ*B is a transcription factor family that regulates the growth and survival of B cells; mantle cell lymphoma cells depend on NF-*κ*B signaling for continued growth and proliferation. The NF-*κ*B signaling pathways are categorized into canonical and non-canonical types, wherein the canonical pathway prompts inflammatory responses, immune regulation, and cell proliferation, while the non-canonical leads to B cell maturation and lymphoid organogenesis. Since these pathways upregulate survival genes and tumor-promoting cytokines, they can be activated to overcome the inhibitory effects of targeted agents, thereby having profound effects on tumorigenesis. The NF-*κ*B pathways are also highly targetable in that they are interconnected with numerous other pathways, including B cell receptor signaling, PI3K/Akt/mTOR signaling, and toll-like receptor signaling pathways. Additionally, elements of the non-canonical NF- *κ*B pathway, such as NF-*κ*B-inducing kinase, can be targeted to overcome resistance to targeting of the canonical NF- *κ*B pathway.

Targeting the molecular mechanisms of the NF-*κ*B pathways can facilitate the development of novel agents to treat malignancies and overcome drug resistance in patients with relapsed or refractory mantle cell lymphoma.

## Background

Mantle cell lymphoma (MCL) is an aggressive B cell lymphoma with one of the worst prognoses of all non-Hodgkin lymphomas. MCL cells are marked by a t(11:14) chromosomal translocation and overexpression of cyclin D1. Although MCL is incurable to date, impressive response rates have been achieved via targeted agents, such as ibrutinib, a Bruton’s tyrosine kinase (BTK) inhibitor. Unfortunately, MCL cells inevitably develop resistance to ibrutinib, making it difficult to treat. While some MCL cell lines are highly sensitive to the B cell receptor (BCR) signaling inhibitors ibrutinib and sotrastaurin, other MCL cell lines, including Z-138 and Maver-1, are insensitive and demonstrate activation of the non-canonical NF-*κ*B pathway, instead of the canonical pathway [1]. This finding suggests that patients with drug-resistant MCL may benefit from alternate treatment approaches, particularly those that are independent of the BCR signaling pathway.

The nuclear factor kappa-light-chain enhancer of activated B cells (NF-*κ*B) is a transcription factor family that regulates the expression of growth factors, cytokines, chemokines, adhesion molecules, and apoptosis inhibitors [2]. NF-*κ*B is known for its regulatory role in inflammatory responses and other pathological processes, including cell differentiation and survival. The NF-*κ*B family has five monomers: RelA, RelB, c-Rel, p50, and 52, which combine to form up to 15 different NF-*κ*B complexes, of which p50–p65 (canonical) and p52-RelB (non-canonical) are paradigmatic.

There are several pathways for NF-*κ*B activation, but the two primary pathways are the canonical and non-canonical pathways. The canonical pathway is triggered by toll-like microbial pattern recognition receptors (TLRs) and pro-inflammatory cytokines such as tumor necrosis factor alpha (TNF*α*) and interleukin-1 (IL-1), which leads to the activation of RelA- or cRel-containing complexes [3]. The non-canonical pathway is activated by TNF (tumor necrosis factor) family cytokines, including lymphotoxin *β* (TNFSF3), CD40 ligand (CD40L and TNFSF5), and B cell-activating factor (BAFF and TNFSF13B). The canonical pathway has downstream effects including inflammatory responses, immune regulation, and cell proliferation, while the non-canonical pathway’s downstream effects lead to B cell maturation and lymphoid organogenesis. An understanding of NF-*κ*B pathway mechanisms in MCL tumorigenesis will facilitate the development of more effective therapeutic agents that suit different patient populations (Table 1).

**Table 1:** Various agents targeting the NF-*κ*B pathway

Agent name	Agent mechanism	Relevant target pathway	Tested in MCL cells/patients?
Ibrutinib	Bruton’s tyrosine kinase (BTK) inhibitor	Canonical NF-*κ*B pathway; BCR signaling	Yes—tested in vitro, in vivo, in clinical trials; approved by the FDA; 68% overall response rate in MCL patients [54]
Acalabrutinib	Second-generation Bruton’s tyrosine kinase (BTK) inhibitor	Canonical NF-*κ*B pathway; BCR signaling	Yes—tested in relapsed or refractory mantle cell lymphoma in a single-arm, multicenter, phase 2 trial; 81% overall response and 40% complete response for 124 patients at a median follow-up of 15.2 months [55]
Bortezomib	Proteasome inhibitor → prevents degradation of ubiquitinated I*κ*B; induces cell death via oxidative and ER stress → NOXA upregulation (NF-*κ*B independent)	Canonical NF-*κ*B pathway	Yes—tested in vitro, in vivo, and in clinical trials; approved by the FDA; 33% overall response rate in R/R MCL patients [56]
Rituximab	Chimeric anti-CD20 antibody; downregulates Bcl-x(L) expression; decreases the phosphorylation of NF-*κ*B-inducing kinase, I*κ*B kinase, and I*κ*Bα; diminishes IKK kinase activity; and decreases NF-*κ*B DNA-binding activity	Canonical and non-canonical NF-*κ*B pathways	Yes—widely used in clinical treatment of patients with non-Hodgkin lymphoma (NHL); also tested in vitro in CD20(+) drug-resistant cell lines Ramos (Bcl-2(−)/Bcl-x(L)(+)) and Daudi (Bcl-2(+)/Bcl-x(L)(+)) [57]
Lenalidomide	Downregulates pro-inflammatory cytokines, such as TNF-α, IL-1, and IL-6	Canonical NF-*κ*B pathway	Yes—approved for the treatment of patients with MCL whose disease has relapsed or progressed after two prior therapies, one of which included bortezomib
Idelalisib	PI3Kδ inhibitor	Cross-talk between NF-*κ*B and PI3K/Akt pathways	Yes—phase I study in 2014 for treatment of relapsed/refractory MCL patients, overall response rate of 40% (16/40 patients) [58]; phase I study in 2014 for treatment of patients with indolent non-Hodgkin lymphoma (NHL), overall response rate of 47% (30/64 patients) [44]
Auranofin	Inhibits homodimerization of toll-like receptor 4 (TLR4), thereby suppressing TLR-mediated activation of NF-*κ*B [59]	Canonical NF-*κ*B pathway; TLR signaling	Phase I/II clinical trial at University of Kansas Medical Center to evaluate safety and efficacy of auranofin in chronic lymphocytic leukemia (CLL), small lymphocytic lymphoma (SLL), and/or prolymphocytic lymphoma (PLL) patients (clinicaltrials.gov)
Duvelisib	PI3K inhibitor	Cross-talk between NF-*κ*B and PI3K/Akt pathways	Yes—tested in vitro and in patient-derived xenograft studies; inhibited MCL growth in vitro and in PDX mice [45]
ACP-319	PI3K inhibitor	Cross-talk between NF-*κ*B and PI3K/Akt pathways	Yes—undergoing phase 1/2 clinical trial in combination with ACP-196 in subjects with B cell malignancies, including MCL (no study results posted yet—clinicaltrials.gov)
AM-0216 and AM-0561	NIK inhibitors	Non-canonical NF-*κ*B pathway	Tested in vitro in multiple myeloma cells; was not possible to do in vivo studies due to poor pharmacokinetic properties, but drug combination may be more promising [35]
ASN002	Syk/jak inhibitor	Canonical NF-*κ*B pathway; BCR signaling	Showed anti-proliferative activity in many cell lines and inhibited tumor growth in a multiple myeloma xenograft model; phase I/II ongoing clinical study [60]
CUDC-907	PI3K/histone deacetylase (HDAC) inhibitor	Canonical NF-*κ*B pathway; BCR and TCR signaling	Yes—inhibits tumor growth of ibrutinib-resistant MCL in vitro and in PDX model [61]; phase I/II trial for relapsed or refractory lymphoma or multiple myeloma (clinicaltrials.gov)
Emetine	I*κ*B*α* phosphorylation inhibitor	Canonical NF-*κ*B pathway	Tested in vitro and in vivo in diffuse large B cell lymphoma cells; induced cell death and demonstrated significant inhibition of tumor growth [62]
Lestaurtinib	I*κ*B*α* phosphorylation inhibitor	Canonical NF-*κ*B pathway	Showed biological and clinical activity in phase 1/2 trial for patients with relapsed or refractory acute myeloid leukemia [63]
Mesalamine	Blocks p65-dependent transactivation	Canonical NF-*κ*B pathway	Not tested in MCL cells; first line agent for treating ulcerative colitis; maintains remission in mild to moderate UC [64]
Fenofibrate	Inhibits the TNF-α/NF-*κ*B axis to induce apoptosis; modulates the expression of anti-apoptotic genes associated with MCL; decreases DNA binding of NF-*κ*B	Canonical NF-*κ*B pathway, cross-talk with TNF signaling	Tested in vitro—decreases growth of Mino, SP53, and Jeko-1 cell lines; induces apoptosis in MCL cell lines Mino and Jeko-1 in vitro; decreases cyclin D1 expression in Mino and SP53 [65]

## Main text

### The canonical pathway

The p50–p65 heterodimer is a transcription factor of the NF-kB family that is bound to and inhibited by IκB. Activation of the canonical pathway leads to proteasomal degradation of IκB, leading to downstream gene expression.

The p50–p65 heterodimer is initially bound to IκB, which prevents the heterodimer from entering the nucleus and enabling gene expression. Lipopolysaccharides (LPSs), tumor necrosis factor alpha (TNF-α), and interleukin-1 (IL-1) activate toll-like receptors (TLR), tumor necrosis factor receptors (TNFR), and interleukin-1 receptors (IL-1R), respectively. Activation of these receptors initiates adapter protein and signaling kinase responses, leading to activation of the IκB kinase (IKK) complex. The IKK complex consists of IKK*α*, IKK*β*, and two IKK*γ* (NEMO) kinases, which phosphorylate I*κ*B on the serine residues S32 and S36, leading to the poly-ubiquitination and proteasomal degradation of I*κ*B [4]. This allows the p50 and p65-RelA heterodimer (a complex from the NF-*κ*B family) to be released into the nucleus to induce gene expression.

#### Interactions with signaling pathways that coordinate with the NF-*κ*B canonical pathway

##### BCR signaling

**Fig. 1:**
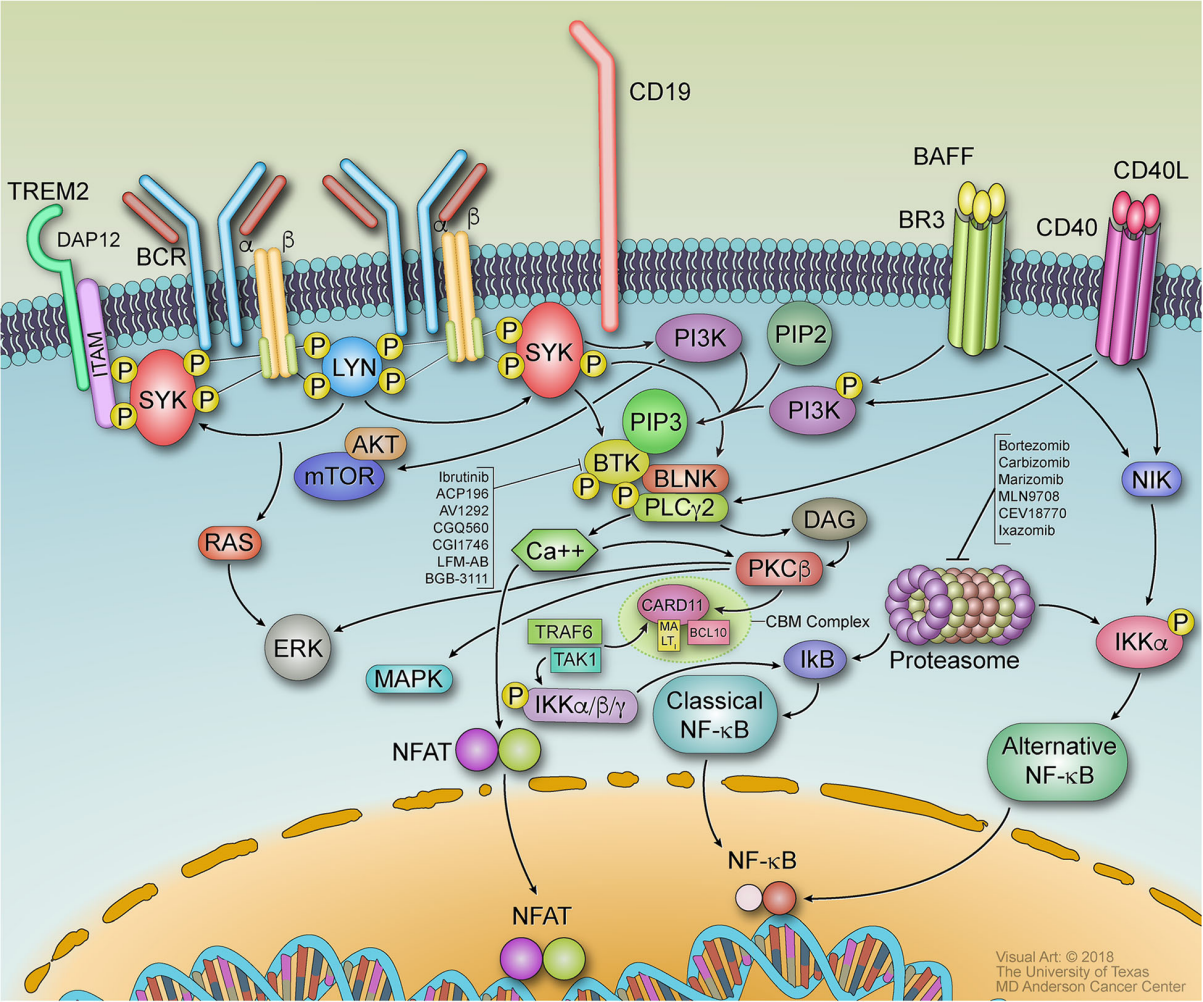
B cell receptor signaling pathway with receptors, inhibitors, targets, and other molecules. B cell receptor signaling mediates the canonical pathway for nuclear translocation of the transcription factor NF-*κ*B. Initial activation of the B cell receptor activates Src family kinases and the Syk and Btk tyrosine kinases, which form a signalosome complex with other signaling enzymes and proteins. Btk phosphorylates and activates PLC*γ*2, which yields the downstream molecules inositol-1,4,5-triphosphate (IP3) and diacylglycerol (DAG) and sensitizes PKCβ due to release of calcium ions. The activated PKC leads to formation of the CBM complex; the IKK complex is then activated, which phosphorylates I*κ*B, allowing it to be ubiquitinated and proteasomally degraded. The p50 and p65-RelA NF-*κ*B heterodimer is then released into the nucleus to induce gene expression

The downstream effects of antigen-mediated BCR signaling lead to activation of BTK and eventually the IKK complex, which leads to gene expression via the canonical pathway. The BCR signaling pathway is mediated by receptor tyrosine kinase-mediated signal transduction. The B cell receptor consists of the immunoglobulins IgM, IgD, Ig-alpha, and Ig-beta, which are expressed by B cells and bound to CD79a/CD79b. When antigens bind to these immunoglobulins, tyrosine kinases including LYN, FYN, and B lymphocyte tyrosine kinase (BLK) phosphorylate the dual-tyrosine containing immunoreceptor tyrosine-based activation motifs (ITAMs) in the cytoplasmic tails of Ig-alpha and Ig-beta [5]. Spleen tyrosine kinase (SYK) binds to the phosphorylated ITAMs and becomes activated, then mediating tyrosine phosphorylation of proteins including B cell linker (BLNK) and B cell adaptor for phosphoinositide 3-kinase (BCAP) (Fig. 1) [6]. Upstream Src family kinases including BLK and LYN, which phosphorylates CD19, activate Bruton’s tyrosine kinase (BTK) [7]. BTK phosphorylates and activates 1-phosphatidylinositol-4,5-bisphosphate phosphodiesterase gamma-2 (PLC*γ*2), which leads to downstream signaling involving Ca^2+^ release and phosphorylation of CARMA/CARD11 by protein kinase C beta (PKCβ) [8]. CARMA/CARD11 associates with B cell lymphoma/leukemia 10 protein (BCL10) and mucosa-associated lymphoid tissue lymphoma translocation protein 1 (MALT1), forming the CARD11-BCL10-MALT1 (CBM) signalosome complex [9]. Once the CBM complex is formed, the IKK complex is activated, leading to p50 and p65-RelA translocation to the nucleus via the canonical NF-*κ*B pathway.

Molecular mechanisms involved in canonical BCR-driven signaling have profound effects on tumorigenesis. For instance, the dysregulation of NF-κB in chronic lymphocytic leukemia (CLL) cells causes overexpression of anti-apoptotic genes; CLL cells have elevated SYK, LYN, and BTK expression and elevated PI3K activity. Ligation of the B cell receptor in vitro has been shown to induce more NF-κB DNA-binding activity in the nucleus of CLL cells, thereby increasing cellular survival [10]. Survival of diffuse large B cell lymphoma (DLBCL) cells and other lymphoma cells similarly depends on CARMA1/CARD11 and NF-*κ*B signaling [11, 12]. Two cell lines of activated B cell-like diffuse large B cell lymphoma (ABC-DLBCL) had high levels of NF-κB DNA-binding activity in the nucleus, constitutive IKK activity, and rapid IκB degradation that were not observed in germinal center B cell-like diffuse large B cell lymphoma (GCB-DLBCL) [11], demonstrating the key role of NF-κB activity in the proliferation of various subtypes of lymphoma cells.

BTK is a major player in initiating the canonical pathway involving BCR signaling. Inhibition of BTK can cause apoptosis in lymphoma cells, which makes BTK a critical therapeutic target [13]. Ibrutinib (PCI-32765) and acalabrutinib, a second-generation BTK inhibitor, effectively block downstream signaling, subsequently inhibiting B cell activation (Table 1) [14–16]. In ABC-DLBCL and Waldenström macroglobulinemia, ibrutinib is highly effective due to the activation of BTK via mutations in CD79B or the myeloid differentiation primary response gene 88 (MyD88), but in mantle cell lymphoma, these mutations are rare [17–19].

To identify what confers ibrutinib sensitivity in mantle cell lymphoma with respect to NF-*κ*B signaling, Saba et al. [17] analyzed the gene expression profiles of 55 tumor samples from 43 previously untreated patients with MCL that were about to undergo therapy. MCL cells of the lymph node demonstrated activation of canonical NF-*κ*B signaling and BCR signaling [17]. Gene expression differed between MCL cells in peripheral blood and lymph nodes because of activation of signaling pathways in the lymph nodes [17]. The ability of tumor cells to proliferate corresponded with the degree of BCR activation.

Saba et al. found that the canonical NF-κB signature with 18 genes dependent on IKKβ activation was on average 2.1-fold more highly expressed in lymph node biopsies than in purified MCL cells (*p* < .0001) [17]. One set of tumors demonstrated dependence on the lymph node environment for BCR and NF-κB activation, whereas another set indicated that BCR and NF-κB genes were not as independent on the lymph node microenvironment [17]. NF-*κ*B-inducing kinase (NIK) signature genes, as markers of the non-canonical NF-κB pathway, were less expressed than the canonical NF-κB signature genes [17]. In MCL cells from the lymph nodes, SYK and p65 were highly phosphorylated, reflecting BCR-dependent activation of the canonical NF-κB pathway [17]. When ibrutinib was administered to inhibit BTK, ibrutinib reduced phosphorylation of p65 and killed 35 to 50% of the tumor cells within 48 h of administration [17]. BCR signaling is activated in the lymph node microenvironment in vivo and appears to promote tumor proliferation and survival, making the canonical pathway a viable target.

##### TLR signaling

Toll-like receptor (TLR) signaling is of particular relevance in mantle cell lymphoma, as TLRs are important proteins involved in the innate immune system. Specifically, increased TLR4 expression in MCL cells can contribute to tumor progression [20]. TLRs recognize pathogen-associated microbial patterns (PAMPs) and danger-associated molecular patterns (DAMPs) to help cells recognize foreign invaders and trigger inflammatory responses. TLR4 activation in patients with recurrent bacterial infections promotes tumor growth and shields MCL cells from surveillance by the immune system [20]. Toll-like receptor signaling is twofold: one pathway involving TLR4 depends on MyD88, mediating early NF-*κ*B activation, and the other pathway depends on TIR-domain-containing adapter-inducing interferon-β (TRIF), mediating late activation of NF-*κ*B.

Both TLR pathways begin with lipopolysaccharide (LPS) binding to LPS-binding protein and forming a complex that binds CD14 to the cell membrane. LPS is then transferred to MD-2 and TLR-4 [21]. TLR-4 subsequently activates the MyD88-dependent pathway with Mal/TIRAP and the TRIF-dependent pathway with TRAM. Unlike IL-1R signaling, MyD88 interacts with Mal for recruitment to the receptor complex [22]; complexes I, II, and III otherwise form in the same manner as that for IL-1R signaling. MyD88 additionally recruits TNF receptor-associated factor 6 (TRAF6) and members of the interleukin-1 receptor-associated kinase (IRAK) family, i.e., IRAK-1, which interact with BCL10 in complex I. BCL10 then binds to Pellino2 and MALT1 (in complexes II and III, respectively), facilitating the activation of TRAF6 [23]. TRAF6, along with Ubc13 and Uev1A, which are ubiquitin-conjugating proteins, activates the TAK1 complex [24]. The TAK1 complex can then activate IKK, triggering the usual cascade of events in the canonical pathway. Targeting TLR-mediated NF-*κ*B signaling may increase the susceptibility of MCL cells to immune surveillance and subsequently minimize tumor progression.

Stimulation of TLR4 signaling via LPS has been found by Wang et al. to increase proliferation of the following MCL cell lines: SP53, Jeko-1, Mino, and Granta-519 [20]. MCL cells expressed many different TLRs, of which TLR4 was one of the most highly expressed [20]. LPS-induced TLR4 signaling also increased NF-*κ*B phosphorylation and activated expression of important cytokines, including interleukin-1 and the vascular endothelial growth factor (VEGF) in MCL cell lines and primary patient cells with TLR4 and MyD88 expression [20]. LPS-mediated TLR4 signaling in MCL cells also facilitated immune evasion by inhibiting T cell proliferation. Cells without TLR4 had a much weaker ability to inhibit T cell proliferation, confirming the key pro-tumor role of TLR4 in MCL cell survival [20].

TLR1/2 and TLR5 have also been found to be expressed in MCL cell lines and primary MCL cells [25]. The activation of TLR2 and TLR5 further activates Akt and MAPK signaling, leading to overexpression of cyclin D1 and D3 and increased proliferation of MCL cells. TLR1/2 and TLR5 activation also affects the canonical NF-*κ*B pathway and enhances the survival and migration of MCL cells [25]. Primary MCL cells have also shown an intermediate response to stimulation with CpG oligodeoxynucleotides, which are detected by TLR9 [26]. TLR9 upregulates the expression of CD20 upon binding with the CpG motif and interacts with BTK to induce B cell proliferation [26, 27].

TLR signaling interplays with BCR signaling mechanisms, affecting the overall survival of MCL patients. Akhter et al. used the NanoString nCounter technology to digitally quantify BCR and TLR signaling molecules in a cohort of 81 MCL patients [27]. This cohort was split into two subsets: those with high BCR activation and those with low BCR activation (> 1.5-fold change in expression, *p* < 0.05) [27]. There was a significant difference in expression of TLR6, TLR7, and TLR9 between the subsets of patients, with fold changes of 2.2, 1.9, and 2.4, respectively (*p* < 0.05 for all) [27]. Overexpression of TLR6 and TLR9 was associated with a poor clinical outcome and worse overall survival in patients with hyperreactive BCR signaling [27]. TLR4 expression was not significantly different between the two subsets of patients, which failed to validate the findings of Wang et al. [20] that MCL cells have high expression of TLR4 [27]. TLR9, on the other hand, was overexpressed in the subset with high BCR activation, in sync with the overexpression of key mediators in the BCR signaling pathway, including BTK, BLNK, and SYK, suggesting that BCR may be activated in a tonic, antigen-independent, or restricted antigen manner [27]. Targeting MCL through TLR inhibitors in combination with other agents targeting NF-*κ*B pathways may be a promising therapeutic choice.

##### TNF-R signaling

NF-*κ*B activation can lead to apoptosis or survival, depending on the apoptotic stimulus. Interestingly, TNF-α signaling causes NF-*κ*B to have anti-apoptotic effects, protecting the proliferating tumor cells; inhibition of NF-*κ*B sensitizes tumor cells to TNF-α-induced apoptosis [28].

Upon activation, a TNF receptor uses its death domain, tumor necrosis factor receptor type 1-associated death domain (TRADD), to bind to RIP1 and TRAF2 [29]. Once the TNF receptor is endocytosed, TRADD detaches from the receptor and associates with another protein: Fas-associated protein with death domain (FADD). The interaction of FADD with caspase-8 activates a caspase cascade, which leads to apoptosis [30]. Meanwhile, TRAF2 ubiquitinates itself and RIP1, bound to TAB2 and NEMO/IKK*γ*, prompting recruitment of TAK1 (which regulates MAP3K3 activity) and IKK*β*. TNF stimulation causes RIP1 to recruit MEKK3/MAP3K3, which phosphorylates IKK*β* [31]. The IKK complex is then activated, leading to the poly-ubiquitination and proteasomal degradation of I*κ*B, allowing the p50–p65 heterodimer to enter the nucleus. RIP1 can also act independently of TAK1 by interacting with p62, which leads to activation of atypical protein kinase C (aPKC) and subsequent activation of the IKK complex [32].

2′-Deoxy-2′-β-fluoro-4′-azidocytidine (FNC) is a cytidine analogue that inhibits proliferation of mantle cell lymphoma cells in vitro and in vivo by inducing apoptosis. Zhang et al. found that administration of FNC to Jeko-1 cells induces apoptosis through the signaling of death receptors, which are members of the TNF superfamily [33]. FNC treatment increased expression of TNF-α, Fas, and the Fas ligand [33]. Upregulation of TNF-α in combination with inhibition of NF-*κ*B activation can increase apoptosis in mantle cell lymphoma cells, presenting a route by which MCL tumors can be targeted.

### The non-canonical pathway

**Fig. 2:**
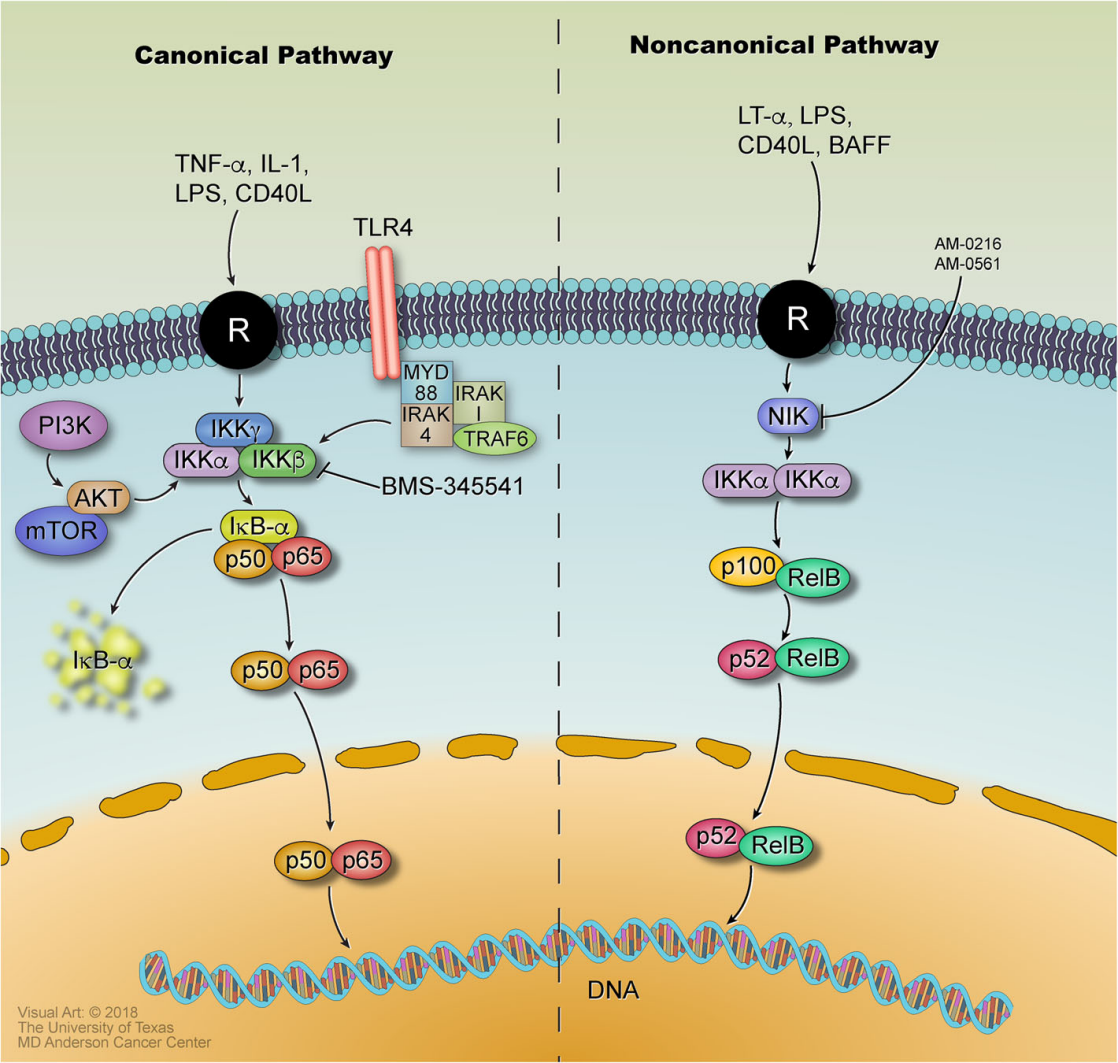
NF-*κ*B signaling pathways with receptors, inhibitors, targets, and other molecules The canonical and non-canonical pathways for NF-*κ*B signaling are mediated by various receptors and signaling molecules, including toll-like receptors (TLR), tumor necrosis factor receptors (TNFR), interleukin-1 receptor (IL-1R), CD40, initiation of B cell activation factor (BAFFR), lymphotoxin β- receptor (LTβR), and receptor activator for nuclear factor kappa B (RANK). The canonical NF-*κ*B pathway involves the inhibition of NF-*κ*B by I*κ*B, which binds to the p50–p65 heterodimer in the cytoplasm and prevents it from entering the nucleus. Activation of BCR, TNFR, and IL-1R receptors initiates adapter protein and signaling kinase responses, leading to activation of the IκB kinase (IKK) complex. Kinases in the IKK complex phosphorylate I*κ*B and lead to its poly-ubiquitination and proteasomal degradation. This allows the p50 and p65-RelA heterodimer (a complex from the NF-*κ*B family) to be released into the nucleus to induce gene expression. In the non-canonical pathway, IKK*α* is activated by the upstream kinase NF-*κ*B-inducing kinase (NIK), which promotes the processing of p100 into the active RelB-p52 isoform of NF-*κ*B. NIK is downregulated by the expression of TRAF2 and TRAF3, which are negative regulators of non-canonical NF-*κ*B signaling that interact with BIRC2 and BIRC3 [1]. Unlike the canonical pathway, the non-canonical pathway does not rely on IKK*β* or IKK*γ* (NEMO); it only needs IKK*α* to phosphorylate the p52 precursor, p100

The non-canonical pathway is activated by initiation of B cell activation factor (BAFFR), CD40, lymphotoxin β-receptor (LTβR), or receptor activator for nuclear factor kappa B (RANK) signaling. The non-canonical NF-*κ*B pathway involves the processing of p100, where p100 is phosphorylated by IKK*α* on the serine residues S866 and S870 and then poly-ubiquitinated (Fig. 2) [34]. This leads to the activation of RelB-p52 complexes, which are heterodimeric subunits of NF-*κ*B. IKK*α* is activated by the upstream kinase NF-*κ*B-inducing kinase (NIK), which promotes the processing of p100 into the active p52 isoform. NIK is downregulated by the expression of TRAF2 and TRAF3, which are negative regulators of non-canonical NF-*κ*B signaling that interact with BIRC2 and BIRC3 [1]. Unlike the canonical pathway, the non-canonical pathway does not rely on IKK*β* or IKK*γ* (NEMO); it only needs IKK*α* to phosphorylate the p52 precursor, p100. Targeting non-canonical signaling mechanisms can overcome resistance to therapies that only target canonical NF-*κ*B signaling.

#### Interactions with signaling pathways that coordinate with the NF-*κ*B non-canonical pathway

##### CD40 signaling

When a CD40 ligand binds to the CD40 receptor on the cell membrane, TRAF proteins are recruited to and directly bind to the CD40 receptor. TRAF proteins negatively regulate NIK. When the NF-*κ*B pathway is inactive, NIK is constantly degraded via ubiquitination by TRAF3. NIK activity is also suppressed by expression of cellular inhibitor of apoptosis 1 (cIAP1) and cellular inhibitor of apoptosis 2 (cIAP2) [35]. However, when the pathway is activated via CD40 ligation, TRAF2 and cIAP 1/2 cause TRAF3 to be proteasomally degraded [36]. NIK can then accumulate with increased stability and phosphorylate IKK*α*, which is required for the phosphorylation and processing of p100 to form p52.

CD40 is expressed in mature B cells, including mantle cell lymphoma cells. While some MCL cells are sensitive to BCR signaling inhibition by ibrutinib, many patients still demonstrate resistance to inhibition of the canonical NF-*κ*B pathway [37]. Culturing the MCL Rec-1 cell line with the CD40 ligand weakened the ability of ibrutinib to inhibit proliferation of Rec-1 cells. The effectiveness of ibrutinib, a BTK inhibitor, was undermined due to how CD40 signaling activates the NF-*κ*B p52 isoform via the non-canonical pathway, which promotes cell survival in opposition to ibrutinib’s inhibitory effects on the canonical pathway [38]. Targeting CD40-mediated NF-*κ*B signaling, in addition to targeting canonical BCR signaling, may help overcome ibrutinib resistance in patient populations with activated CD40-CD40L pathways [38].

##### BAFFR signaling

B cell-activating factor receptor (BAFFR) signaling mechanisms are similar to those of CD40 signaling. BAFFR is a member of the TNFR family, and it predominantly activates the non-canonical NF-*κ*B pathway [39]. BAFFR can interact with TRAF3 but not TRAF2, which is why BAFFR cannot trigger the canonical pathway. Degradation of TRAF3 triggers non-canonical NF-*κ*B signaling [40]. This leads to p100 processing via NIK activation and the same downstream molecular interactions as with CD40 signaling. BAFFR may be a key biomarker of both normal and abnormal B cells, especially due to its role in activating the non-canonical NF-*κ*B pathway. Targeting BAFFR-mediated NF-*κ*B signaling may offer a novel approach in suppressing neoplastic B cell maturation and proliferation.

### Interaction with the PI3K/AKT pathway

NF-*κ*B signaling is also mediated through interactions with other pathways, such as the PI3K/Akt pathway. In Burkitt’s lymphoma, an aggressive form of non-Hodgkin lymphoma, NF-*κ*B activation and STAT3 activation depend on upstream signaling of phosphatidylinositol 3-kinase (PI3K), which plays a key role in survival signaling [41]. Inhibition of PI3K blocks interleukin-1 signaling (IL-1), preventing eventual translocation of the p65-RelA heterodimer to the nucleus. Lipid products of PI3K activate the serine/threonine kinase Akt/PKB, which mediates cell survival and proliferation [42]. While Akt does not directly phosphorylate NF-*κ*B, it affects the canonical NF-*κ*B signaling pathway by phosphorylating IKKα, which targets the IκB inhibitor protein and allows NF-*κ*B to translocate to the nucleus [42]. Overexpression of IκB conversely interferes with the ability of PI3K and Akt to induce oncogenic transformation, indicating that the PI3K/Akt and NF-*κ*B pathways depend on one another.

Through gene expression profiling, several genes in mantle cell lymphoma cells have been identified that relate to the PI3K/Akt pathway; the following genes were found to be altered or upregulated: PIK3CA, PDK2, PDPK1, AKT1, RPS6KB2, FOXO3A, PPP2R2C, and PDK1 [43]. The PI3K/Akt pathway may confer resistance to apoptosis in MCL cells. PI3K inhibitors such as duvelisib and idelalisib have been found to have anti-tumor activity in relapsed MCL [44, 45]. PI3K/Akt survival signaling is supplemented by the survival signaling of NF-*κ*B, suggesting that targeting both pathways in a joint manner may be highly effective in limiting MCL cell proliferation and preventing tumor growth in patients.

### Relevance of NF-*κ*B pathways for the treatment of MCL

Mantle cell lymphoma does not have any single clear oncogenic driver and is heterogeneous, characterized by mutations in genes including ATM, CCND1, UBR5, TP53, BIRC3, NOTCH1/2, and TRAF2 [46]. Mutations in elements of the canonical NF-*κ*B pathway, such as the CBM complex or IKK-beta, cause activation of the canonical pathway. This allows drugs such as ibrutinib and acalabrutinib, BTK inhibitors, to effectively inhibit BCR signaling and suppress growth in cells sensitive to BCR signaling inhibition [47]. MCL cells that are resistant to BCR signaling inhibition tend to have somatic mutations in inhibitors of the non-canonical NF-*κ*B pathway, such as cIAP1, cIAP2, and TRAF2/3; these mutations cause resistant cell lines (Z-138, Maver-1) to depend on the deregulated non-canonical NF-*κ*B pathway for survival and proliferation (Fig. 2).

When PKC-B, an important kinase upstream of the CBM complex in BCR signaling, was depleted via sh-RNA, proliferation of the ibrutinib-sensitive MCL cell line Jeko-1 was suppressed, while the insensitive cell line Granta-519 was left unaffected. This indicates that a subset of cell lines strongly depends on canonical NF-*κ*B signaling. Treatment with sotrastaurin (STN) was also found to selectively modulate I*κ*B*α* phosphorylation and reduce RelB cleavage, which is a marker of CBM complex activity, in STN-sensitive MCL cell lines [48]. In addition, the CBM complex component CARD11 appeared to be highly expressed in sensitive MCL lines, suggesting that BCR pathway components can be deregulated to treat cells that are sensitive to inhibition of the canonical NF-*κ*B pathway [49].

MCL tumors can also be targeted via other pathways that interact with NF-*κ*B signaling, for instance, through the PI3K /Akt pathway, CD40 signaling, BAFFR signaling, or transglutaminase (TG2) signaling. The expression of transglutaminase (TG2), a calcium-dependent protein encoded by the TGM2 gene associated with tumor cell proliferation, metastasis, and drug resistance, is closely linked with constitutive activation of NF-*κ*B [50]. Upregulation of TG2 expression increased IL6 expression 1.8- to 2.9-fold and stimulated autophagy formation, a protective mechanism for tumor cells [50]. In comparison with normal B cells, patients with a blastoid type of MCL, an aggressive variant with a worse overall survival, displayed elevated TGM2 levels with up to 150-fold increases; these blastoid MCL subtypes also had higher TGM2 levels than classical MCL [50]. By silencing TG2 via CRISPR/Cas9, Zhang et al. observed that p53, p21, and p27 levels increased and cyclin gene levels decreased, indicating cell cycle arrest, levels of anti-apoptotic genes including BCL-XL and BCL-2 decreased, and levels of pro-apoptopic genes including BAX, BAK, and NOXA increased [50]. NF-*κ*B p50 and p65 DNA-binding activity, downstream activation of IL8, p-STAT3 expression, and IL6 levels were significantly decreased in TG2 knockout MCL cells whereas signaling activity increased in TG2 overexpression cells [50]. TG2 silencing also conferred sensitivity to chemotherapeutic drugs whereas cells overexpressing TG2 exhibited drug resistance with higher IC50 values [50]. In patients with bortezomib resistance, TG2 signaling can be inhibited by a calcium blocker such as perillyl alcohol and administered in combination with bortezomib to suppress NF-*κ*B expression and improve MCL cell sensitivity to bortezomib [51]. Inhibiting autophagy in MCL cells via TG2 silencing may thus be a promising therapeutic choice to overcome chemotherapy resistance.

Many targeted therapies have focused on targeting B cell receptor signaling in MCL cells, which indirectly reduces canonical NF-*κ*B signaling. Direct inhibitors of NF-*κ*B are scarce, but more targeted therapies are focusing on inhibition of non-canonical signaling and cross-talk with other pathways, such as the PI3K/Akt pathway to overcome resistance to inhibitors of the canonical pathway. For instance, the combination of TGR-1202, a PI3K delta inhibitor, with ibrutinib had an overall response rate of 67% with six out of nine patients achieving a partial response in a phase I/Ib multicenter trial for patients with relapsed/refractory MCL [52]. Other combination therapies that target both canonical and non-canonical pathways have also been effective in inhibiting MCL cell growth and proliferation (Table 2). For instance, the combination of CC-292 with NIK inhibitors, AM-0216 and AM-0561, in Z138 and MAVER-1, cell lines resistant to CC-292 and ibrutinib, resulted in a significant decrease in p52 levels, via inhibition of the non-canonical pathway, and a complete lack of IκB phosphorylation, indicating total inhibition of the NF-*κ*B pathway [53]. This combination was also effective in primary MCL cells with BIRC3 inactivation and is a promising therapeutic choice for further investigation in vivo and in the clinical setting [53].

**Table 2:** Combination therapies targeting the NF-*κ*B pathway

Combination therapy	Target pathway and mechanism	Tested in MCL cells/patients?
Ibrutinib with rituximab	Canonical and non-canonical NF-*κ*B pathways; inhibits BTK; rituximab decreases phosphorylation of NIK, I*κ*B kinase, and I*κ*Bα; diminishes IKK kinase activity; and decreases NF-*κ*B DNA-binding activity	Yes; ongoing phase II trial at the MD Anderson Cancer Center of rituximab in combination with ibrutinib in relapsed/refractory MCL (clinicaltrials.gov)
Thalidomide with rituximab	Canonical and non-canonical NF-*κ*B pathway; thalidomide inhibits IKK and reduces TNF-α production, along with effects of rituximab	Yes—thalidomide combined with rituximab has antitumor activity in relapsed/refractory MCL; 81% overall response rate to rituximab plus thalidomide [66]
Lenalidomide with rituximab	Canonical and non-canonical NF-*κ*B pathway; downregulates pro-inflammatory cytokines, such as TNF-α, IL-1, and IL-6, along with effects of rituximab	Yes—overall response rate of 87% when combined with rituximab in MCL patients [67]
TGR-1202 with ibrutinib	Cross-talk between NF-*κ*B and PI3K/Akt pathways; TGR-1202 inhibits PI3K Delta	Yes—tested in relapsed or refractory MCL and CLL patients in combination with ibrutinib in a phase 1/1b study; overall response rate of 85% in combination with ibrutinib (11/13) [52]
Perillyl alcohol (calcium blocker) with bortezomib	Cross-talk between NF-*κ*B and TG2 signaling; inhibition of autophagy to improve sensitivity to bortezomib	Not tested in patients but tested in MCL cells; was found to suppress NF-*κ*B signaling and improve cytotoxicity of bortezomib [51]
CC-292 with lenalidomide and NIK inhibitors, AM-0216 and AM-0561	Canonical and non-canonical NF-*κ*B pathway; CC-292 inhibits BTK in a highly selective manner; lenalidomide downregulates pro-inflammatory cytokines, such as TNF-α, IL-1, and IL-6; NIK inhibitors inhibit alternative NF-*κ*B signaling	Not tested in patients, but tested in MCL cell lines and primary cells; CC-292 significantly reduced BTK phosphorylation and its activity was enhanced by lenalidomide co-treatment; combination of CC-292 with NIK inhibitors had a significant cooperative effect that inhibited cell growth and induced apoptosis in Z138 and MAVER-1 [53]

## Conclusions

Overall, the NF-*κ*B pathways have numerous molecular mechanisms that lead to the eventual expression of NF-*κ*B target genes. These genes prompt inflammatory responses, immune regulation, and cell proliferation via the canonical pathway and B cell maturation and lymphoid organogenesis via the non-canonical pathway. The multifaceted NF-*κ*B pathways play a crucial role in the growth and proliferation of mantle cell lymphoma cells. Drugs that target various components of the NF-*κ*B pathway have the potential to treat MCL, depending on the pathway to which the cells are sensitive. Targeting NF-*κ*B mechanisms may prove to be a valuable tool in the development of targeted agents to overcome drug resistance and can lead to effective therapies for mantle cell lymphoma.

## Abbreviations

ABC-DLBCL: Activated B cell-like diffuse large B cell lymphoma
BAFF/TNFSF13B: B cell-activating factor
BAFFR: B cell-activating factor receptor
BCAP: B cell adaptor for phosphoinositide 3-kinase
BCL10: B cell lymphoma/leukemia 10 protein
BCR: B cell receptor
BLK: B lymphocyte tyrosine kinase
BLNK: B cell linker protein
BTK: Bruton’s tyrosine kinase
C40L/TNFS5: CD40 ligand
CBM: CARD11-BCL10-MALT1 signalosome complex
cIAP1: Cellular inhibitor of apoptosis 1
cIAP2: Cellular inhibitor of apoptosis 2
CLL: Chronic lymphocytic leukemia
DAG: Diacylglycerol
DAMP: Danger-associated molecular patterns
DLBCL: Diffuse large B cell lymphoma
ER: Endoplasmic reticulum
FADD: Fas-associated protein with death domain
FNC: 2′-Deoxy-2′-β-fluoro-4′-azidocytidine
GCB-DLBCL: Germinal center B cell-like diffuse large B cell lymphoma
HDAC: Histone deacetylase
IKK: IκB kinase
IKK*α*: IκB kinase
IKK*β*: IκB kinase
IKK*γ*: IκB kinase
IL-1: Interleukin-1
IL-1R: Interleukin-1 receptor
IP3: Inositol-1,4,5-triphosphate
IRAK: Interleukin-1 receptor-associated kinase
ITAM: Immunoreceptor tyrosine-based activation motifs
LPS: Lipopolysaccharide
LTβR: Lymphotoxin β-receptor
MALT1: Mucosa-associated lymphoid tissue lymphoma translocation protein 1
MCL: Mantle cell lymphoma
MyD88: Myeloid differentiation primary response gene 88
NF-*κ*B: Nuclear factor kappa-light-chain enhancer of activated B cells
NIK: NF-*κ*B-inducing kinase
PAMP: Pathogen-associated microbial patterns
PCI-32765: Ibrutinib, an inhibitor of Bruton’s tyrosine kinase
PI3K: Phosphatidylinositol 3-kinase
PKCβ: Protein kinase C beta
PLC*γ*2: 1-Phosphatidylinositol-4,5-bisphosphate phosphodiesterase gamma-2
RANK: Receptor activator for nuclear factor kappa B
STN: Sotrastaurin
SYK: Spleen tyrosine kinase
TG2: Transglutaminase
TLR: Toll-like microbial pattern recognition receptors
TLR1: Toll-like receptor 1
TLR2: Toll-like receptor 2
TLR4: Toll-like receptor 4
TLR5: Toll-like receptor 5
TLR9: Toll-like receptor 9
TNFR: Tumor necrosis factor receptor
TNFSF3: Lymphotoxin *β*
TNF*α*: Tumor necrosis factor alpha
TRADD: Tumor necrosis factor receptor type 1-associated death domain protein
TRAF: TNF receptor-associated factor
TRIF: TIR-domain-containing adapter-inducing interferon-β
VEGF: Vascular endothelial growth factor
